# HIV psychiatry in the era of combined antiretroviral therapy: top five issues

**DOI:** 10.7448/IAS.18.1.20725

**Published:** 2015-11-06

**Authors:** Jorge L Zirulnik, Héctor M Perez

**Affiliations:** HIV/AIDS Infectology Division, JA Fernandez Hospital, Buenos Aires, Argentina

Since the introduction of combined antiretroviral therapy (cART) regimens in the middle of the 1990s, HIV infection has turned into a chronic manageable disease instead of the death sentence it used to be. Nowadays, with the expansion of this kind of highly active therapy, the survival rate is almost equal to that of the general population [1,2].

It has been 30 years since the concept of the AIDS dementia complex was introduced to the field of psychiatry. A group led by Navia, Jordan and Price described it in 1986, a decade before the emergence of highly active antiretroviral treatment. Since then, there has been significant evolution in diagnostics and nosology. Progress was also achieved in therapeutic strategies, especially with the use of psychotropic drugs but also through the increased awareness of the psychological needs of people living with HIV. These changes have led HIV psychiatrists to work in a complex and fascinating bio-psychosocial interface. In this article, we present and discuss the five main points that are critical in HIV-related psychiatric contexts. These include the emerging model of HIV-related psychiatric assistance; the impact of highly active antiretroviral therapy on HIV infection status and survival, in the context of heavy, longstanding psychosocial stigma; the drastic decrease in the prevalence of HIV dementia; psychotropic treatment strategies and their interactions with antiretroviral drugs; and lastly the application of psychotherapy techniques within HIV settings [3].

Firstly, the classic model of mental healthcare of HIV patients, namely *consultation-liaison* psychiatry, has evolved into a new transdisciplinary field. Consultation-liaison psychiatry traditionally separates its services, leading to bureaucratic procrastination, late diagnosis or even malpractice. This model also introduces unnecessary confusion for the health professionals involved, particularly in relation to specific HIV issues such as the potential interactions between psychotropic drugs and antiretrovirals or the psychological factors contributing to low adherence. We believe that this first model is now obsolete. The current model, which we call *in setting dispositive*, is increasingly implemented in many HIV patient units, especially in the developed world. In this model, psychiatrists work next to infectious disease specialists and other relevant clinicians. As a result, excellence in HIV mental health treatment can be reached [4,5].

Secondly, cART regimes have brought about drastic changes in HIV infection since 1996. The disease has become a manageable chronic condition, albeit with significant demo-epidemiological and psychosocial consequences. People with HIV live longer than before, although the stigma remains. Despite scientific advances in the biological dimension of the illness (including far less incidence of opportunistic infections and vertical transmission), there are still negative psychosocial conditions for people living with HIV. Unfortunately, the discrimination or exclusion that HIV-positive people deal with, at the workplace for example, increases their risk of suffering from depression, which is the most common disorder in this field. This disorder has alarming prevalence figures, exceeding even 35% in some settings. HIV depression should be taken as one of the diagnostic standards in all HIV care clinics. Neglecting detection and treatment of HIV depression has potentially negative consequences, causing, for example, a person with HIV to stop his or her antiretroviral treatment [6,7].

Thirdly, in the pre-cART era (1981 to 1996), HIV was shown to have neurological effects capable of leading to dementia in infected people. In some HIV settings, prevalence of dementia was detected in 30% of HIV-positive people. Dementia is characterized by a primary neuropathogenesis that results from the deleterious action of the neurotoxic proteins of the virus on the central nervous system, rather than direct neuronal damage. With the advent of cART, there was a dramatic decrease, to less than 8%, in the prevalence of HIV-associated dementia. However, according to the recent literature, HIV-associated neurocognitive deficit (HAND) remains prevalent despite efficacy of antiretroviral treatment. HAND in HIV-infected patients who are receiving cART often goes undetected due to the lack of evident clinical manifestation or the difficulty of getting appropriate neuropsychological evaluations. Development of screening instruments that are as sensitive and specific as the International HIV Dementia Scale (also known as the *Sacktor test*) are urgent. These can be used in less than 20 minutes and have the capacity to detect all clinical forms of HAND [8–11].

Fourth, for HIV psychiatrists, one of the most important issues is the proper application of psychopharmacological strategies. This issue can become critical if the patient has a serious mental illness, such as schizophrenia, bipolar disease or borderline personality disorder, in which the use of psychotropic drugs is usually permanent. In such cases, the psychiatrist should know the potential pharmacokinetic drug interactions between psychotropic and antiretroviral drugs, particularly if the patient is receiving protease inhibitors (PIs) such as ritonavir or cobicistat – both of which exert a high inhibitory power on the hepatic cytochrome P450 system. The emergence of PI-free drug regimens, based on integrase inhibitors, have significantly facilitated the psychopharmacological treatment of patients with serious psychiatric comorbidities [12,13].

Lastly, more research is urgently needed in the psychiatric HIV field particularly around emerging challenges such as the impact of the use of techniques of intensive or ultra-short cognitive-behavioural therapy, for example. This will be useful in addressing any psychologically based adherence problems, as well as gaining a better understanding of the HAND mechanism and of new therapeutic approaches (in addition to the penetration of antiretroviral drugs in the central nervous system).

More randomized controlled trials conducted with HIV patients on psychotropic drugs, especially antidepressants and antipsychotics, are needed to better understand their use in real-life clinical practice. Finally, research is required on effective psychoeducational approaches to best contribute to the fight against community exclusion or stigmatization of people living with HIV.
